# Risk Stratification in Transthyretin Cardiac Amyloidosis: The Added Value of Lung Spirometry

**DOI:** 10.3390/jcm12113684

**Published:** 2023-05-26

**Authors:** Rishika Banydeen, Reid Eggleston, Antoine Deney, Astrid Monfort, Jay H. Ryu, Giuseppe Vergaro, Vincenzo Castiglione, Olivier Lairez, Michele Emdin, Jocelyn Inamo, Misbah Baqir, Remi Neviere

**Affiliations:** 1 Department of Clinical Research, CHU Martinique (University Hospital of Martinique), 97200 Fort de France, France; rishika.banydeen@chu-martinique.fr; 2 Cardiovascular Research Team EA7525, Université des Antilles (University of the French West Indies), 97200 Fort de France, France; astrid.monfort@chu-martinique.fr (A.M.); jocelyn.inamo@chu-martinique.fr (J.I.); 3 Division of Pulmonary and Critical Care Medicine, Mayo Clinic School of Graduate Medical Education, Mayo Clinic College of Medicine and Science, Mayo Clinic, Rochester, MN 55905, USAbaqir.misbah@mayo.edu (M.B.); 4 Department of Cardiology, Rangueil Hospital, CHU Toulouse (University Hospital of Toulouse), 31400 Toulouse, France; 5 Department of Cardiology, CHU Martinique (University Hospital of Martinique), 97200 Fort de France, France; 6 Mayo Clinic School of Graduate Medical Education, Mayo Clinic College of Medicine and Science, Mayo Clinic, Rochester, MN 55905, USA; 7 Fondazione Toscana Gabriele Monasterio per la Ricerca Medica e di Sanita Pubblica, 56124 Pisa, Italy; 8 Institute of Life Sciences, ScuolaSuperioreSant’Anna, Pisa, Italy & Fondazione Toscana Gabriele Monasterio, 56124 Pisa, Italy

**Keywords:** transthyretin cardiac amyloidosis, prognosis, forced vital capacity, lung volume restriction, cardiopulmonary exercise, biomarker staging, adverse outcome

## Abstract

Transthyretin cardiac amyloidosis (ATTR-CA) is an increasingly recognized disease that often results in heart failure and death. Traditionally, biological staging systems are used to stratify disease severity. Reduced aerobic capacity has recently been described as useful in identifying higher risk of cardiovascular events and death. Assessment of lung volume via simple spirometry might also hold prognostic relevance. We aimed to assess the combined prognostic value of spirometry, cardiopulmonary exercise testing (CPET) and biomarker staging in ATTR-CA patients in a multi-parametric approach. We retrospectively reviewed patient records with pulmonary function and CPET testing. Patients were followed until study endpoint (MACE: composite of heart-failure-related hospitalization and all-cause death) or censure (1 April 2022). In total, 82 patients were enrolled. Median follow-up was 9 months with 31 (38%) MACE. Impaired peak VO_2_ and forced vital capacity (FVC) were independent predictors of MACE-free survival, with peak VO_2_ < 50% and FVC < 70% defining the highest risk group (HR 26, 95% CI: 5–142, mean survival: 15 months) compared to patients with the lowest risk (peak VO_2_ ≥ 50% and FVC ≥ 70%). Combined peak VO_2_, FVC and ATTR biomarker staging significantly improved MACE prediction by 35% compared to ATTR staging alone, with 67% patients reassigned a higher risk category (*p* < 0.01). In conclusion, combining functional and biological markers might synergistically improve risk stratification in ATTR-CA. Integrating simple, non-invasive and easily applicable CPET and spirometry in the routine management of ATTR-CA patients might prove useful for improved risk prediction, optimized monitoring and timely introduction of newer-generation therapies.

## 1. Introduction

Transthyretin cardiac amyloidosis (ATTR-CA) is an increasingly recognized condition which originates primarily from the abnormal extracellular accumulation of insoluble misfolded ATTR protein deposits within the myocardial interstitium [1]. Two types of ATTR amyloid fibrils are distinguished: non-mutated (ATTRwt) or variant transthyretin (ATTRv). 1,2 Progressive cardiac amyloid fibril accumulation is synonymous with restrictive heart wall chamber behavior and impaired myocardial contractile reserve, which often lead to symptomatic chronic heart failure and death [1,2].

Traditional clinical echocardiographic parameters and cardiac biomarkers have been accurately used to stratify disease severity in ATTR-CA patients [2,3,4]. Biological staging systems applicable to both wild-type and variant ATTR-CA, as well as echocardiographic parameters, have been described as having prognostic value [4]. Besides such well-established risk models, several authors have brought to light the pertinence of functional evaluation, such as peak aerobic capacity (pVO_2_) and pulmonary function test, in identifying CA patients with poor prognosis [5,6,7,8,9,10]. The cardiopulmonary exercise test (CPET) is the gold standard test to determine prognosis in chronic heart failure patients with reduced ejection fraction. We and other groups have suggested that the combination of reduced peak VO_2_ and NT-proBNP levels represents a valuable predictor of all-cause mortality and heart-failure-related hospitalization in ATTR-CA patients [5,6,7,10]. While being a safe and useful diagnostic tool in many pathological conditions, CPET requires sophisticated and expensive devices, as well as specialized medical staff, which may limit its clinical use, notably in low-resource settings. In contrast, spirometry is a simple method to evaluate pulmonary function, which is successfully used to stratify heart failure severity [11,12,13]. The use of spirometry to screen for ventilatory defects in patients with heart failure is known to improve risk stratification based on pVO_2_ [13].

While restrictive spirometry pattern is a frequent observation in patients with ATTR-CA [8,9], the objective of the present study was to assess the potential link between the occurrence of major adverse cardiac events and impaired cardiopulmonary function (reduced pVO_2_ and lung volume restriction) in ATTR-CA patients. We also analyzed the prognostic value of these two functional parameters in predicting adverse outcome, and quantified their added predictive ability when combined with a well-established validated prognostic score (ATTR biomarker staging). To the best of our knowledge, the combined prognostic value of these three markers has not yet been documented in ATTR-CA.

## 2. Materials and Methods

### 2.1. Study Context, Design and Population

This multicenter observational study involves four referral centers for cardiac amyloidosis (CA) management, namely the University Hospital of Martinique (Fort de France, France), University Hospital of Toulouse (Toulouse, France), the Fondazione Toscana G. Monasterio (Pisa, Italy) and the Mayo Clinic (Rochester, MN, USA). A retrospective review of the medical records of ATTR-CA patients was conducted.

Inclusion criteria were the conduct of pulmonary function (PFT) and cardiopulmonary exercise testing (CPET) whilst patients were in a stable condition at the four pre-cited expert centers from 1 August 2005 to 23 December 2021. A stable condition was defined as the absence of hospitalization for cardiac decompensation or any other cause in the six months prior to PFT and CPET. Patient exclusion criteria were the age below 18 years, pregnancy, breastfeeding, history of malignancy (other than AL amyloidosis), AL amyloidosis, serum amyloid A amyloidosis, chronic obstructive pulmonary disease, persistent asthma, positivity for human immunodeficiency virus, active hepatitis infection, pre-existing heart diseases and other causes of heart diseases (ischemic, hypertensive, valvular heart disease), or any other concurrent medical condition or disease that would likely interfere with study procedures or results.

The study was approved by the institutional review boards of the participating centers. All patients were managed in accordance with the amended Declaration of Helsinki (https://www.wma.net/policies-post/wma-declaration-of-helsinki-ethical-principles-for-medical-research-involving-human-subjects) (accessed on 25 April 2023). Written informed consent from the patients or patients’ legal guardian/next of kin was not required to participate in this study in accordance with the current legislation and the institutional requirements. Informed consent was obtained from all patients (University Hospital APHP IRB: 00006477, 1 May 2022).

### 2.2. Diagnosis of ATTR Amyloidosis

All participants underwent a thorough clinical, imaging and biological evaluation for ATTR amyloidosis diagnosis [2,14,15] including 12-lead electrocardiography and blood tests such as creatinine, cardiac troponin T (hs) and NT-proBNP. Echocardiography examination was performed in accordance with the recommendations from the American Society of Echocardiography. Cardiac involvement was evoked on ventricular hypertrophy, and a decrease in longitudinal global strain with abnormal apical texture was characterized as a speckled appearance. ATTR amyloidosis was diagnosed by cardiac uptake on the 99mTc-labeled phosphate bone scintigraphy in the absence of monoclonal gammopathy or abnormal free light chains in blood and urine. ATTR amyloidosis diagnosis was confirmed by histological demonstration of amyloid fibrils in salivary duct glands, subcutaneous adipose tissue or endomyocardial biopsies. Genetic testing for transthyretin mutation was performed in all ATTR-CA patients. Gillmore’s validated three-stage biomarker staging score [15] was calculated for each patient according to the NT-proBNP level (cut-off 3000 ng/L) and estimated glomerular filtration rate (cut-off 45 mL/min/m²). Stage I was defined as NT-proBNP ≤ 3000 ng/L and eGFR ≥ 45 mL/min, Stage III was defined as NT-proBNP > 3000 ng/L and eGFR < 45 mL/min, and the remainder of the patients were Stage II.

### 2.3. Pulmonary Function Testing (PFT)

PFT included spirometry, functional residual capacity and total lung volume, and lung diffusion capacity, which was performed on subjects in a sitting position using the guidelines of the European Respiratory Society (ERS) [16]. For study analysis, standard spirometry parameters were considered, which included absolute and percent-of-predicted normal FEV1 (forced expiratory volume in the first second) and FVC (forced vital capacity), as well as the FEV1/FVC ratio. Spiromery was considered as normal (FEV1/FVC ≥ 0.70 and FVC ≥ 80% of predicted values) or restricted (FEV1/FVC ≥ 0.70 and FVC < 80% predicted values) using race-based GLI (Global Lung Function Initiative) predicted values [17,18].

### 2.4. Cardiopulmonary Exercise Testing (CPET)

CPET was performed according to the standardized procedures using an upright electromagnetic braked cycle ergometer as recommended by the American Thoracic Society (ATS) guidelines [19]. Exercise testing contraindications included unstable cardiovascular diseases, orthopedic impairment compromising exercise performance and mental impairment leading to inability to cooperate.

The patients underwent a cardiopulmonary exercise test previously performed as “familiarization” in order to achieve suitable results. The test exercise protocol involved an initial 3 min rest period, followed by unloaded cycling for 2 min with a progressive 5- to 10-watt increment every minute until exhaustion at a pedaling frequency of 60–65 revolutions/minute (rpm). Such incremental phases lasting 8–12 min are efficient and provide suitable information, in particular at peak exercise. Subjects were continuously monitored using 12-lead ECG. Blood pressure was recorded every 2 min. Breath-by-breath cardiopulmonary data were measured at rest, warm-up and during incremental exercise testing. Subjects respired through an oro-nasal mask. Before each test, oxygen (O_2_) and carbon dioxide (CO_2_) analyzers and flow mass sensor were calibrated using the available precision gas mixture and a 3 L syringe, respectively. Minute ventilation (VE), oxygen uptake (VO_2_) and carbon dioxide output (VCO_2_) were recorded as concurrent 10 sec moving averages, which was the determined ventilation anaerobic threshold by the V-slope method.

Peak values were averaged over the last 30 sec of exercise. Peak oxygen pulse (O_2_ pulse), a surrogate of stroke volume, was calculated and expressed in mL per beat and as a percentage of the predicted value by dividing the predicted peak VO_2_ by the predicted peak heart rate (HR). Tidal volume and breathing frequency were measured online. Ventilatory reserve was calculated as (MVV-peak VE)/MVV ∗ 100, where MVV is the maximal voluntary ventilation estimated as FEV1 multiplied by 35 [19]. Ventilatory efficiency, as indicated by the increment in VE relative to VCO_2_ (VEVCO_2_ slope), was calculated offline as a linear regression function using 10 s averaged values [19]. The participants were encouraged to continue on their exercise bout until a true symptom-limited exhaustive level was achieved. Patient effort was considered maximal if two of the following conditions were achieved: predicted maximal work, age-predicted maximal heart rate (HRmax), ventilatory O_2_ equivalent VE/VO_2_ > 45 and respiratory exchange ratio (RER, i.e., volume of carbon dioxide produced/volume of oxygen consumed > 1.10) [19]. Symptoms and subjective ratings of perceived exertion were recorded in order to estimate exertion level.

### 2.5. Follow-Up and Endpoints

The study’s primary endpoint was the occurrence of a major adverse cardiac event (MACE) defined as the composite of heart-failure-related hospitalization or all-cause death. Patient follow-up was carried out from the time of spirometry/CPET until either MACE onset or censoring on 1 April 2022.

### 2.6. Statistical Analysis

Baseline patient, PFT and CPET characteristics were described and compared according to MACE occurrence during follow-up. For all descriptive and inferential analyzes, the assumption of normal data distribution was analyzed. Mean and standard deviations were reported for normally distributed variables and median and interquartile range (IQR) for non-normally distributed variables. Categorical variables were presented as absolute values and percentages. The following tests were used for group comparisons: Student *t*-test, Wilcoxon–Mann–Whitney test, Chi-square test and Fisher’s exact test. Univariate and multivariate logistic regression models were first fitted to assess the independent effect of predictors on MACE occurrence. Receiver Operating Characteristic (ROC) Curve analysis was used to establish the optimal cut-off points for the outcome prediction by the relevant PFT and CPET variables. Time-to-event data were also evaluated with the use of Kaplan–Meier estimates and Cox proportional hazards methods. Due to the relatively small number of clinical events (MACE), the number of variables in multivariate regression models was restrained in accordance with the results of univariate logistic (*p* < 0.05) or Cox regression analysis (*p* < 0.15), as well as the clinical relevance of variables and collinearity. Variables fulfilling the latter conditions were retained for backward stepwise multivariate logistic or Cox regression analysis. The goodness-of-fit of final multivariate models was ascertained. In order to assess the added predictive ability of pertinent pulmonary function testing (PFT) and cardiopulmonary exercise testing (CPET) variables when combined with a well-established validated prognostic score (ATTR biomarker staging) 1, three metric measures were computed: Areas Under the ROC Curve (dichotomous and time-dependent analysis), Integrated Discrimination Improvement (IDI) and Net Reclassification Improvement (NRI). Areas Under the ROC Curve (AUC), IDI and NRI are complementary measures used to quantify improvement in a model’s performance in predicting risk when new markers are added to the existing models [20,21,22]. AUC is a measure of model discrimination (i.e., how well the model separates the subjects who did and did not experience an event). It essentially depicts a tradeoff between the benefit of a model (true positive or sensitivity) vs. its costs (false positive or 1-specificity). AUC ranges from 0.5 (no discrimination) to 1 (perfect discrimination). While the difference in AUC is a common method to compare two models, it is relatively insensitive to detecting clinically important risk differences (1, 2, 3, 7). IDI measures the new model’s improvement in average sensitivity (true positive rate) without sacrificing its average specificity (true negative rate). In comparing the models, IDI measures the increment in the predicted probabilities for the subset experiencing an event and the decrement for the subset not experiencing an event. This adds an important element that AUC lacks. The AUC is a rank-based statistic in which all that matters is which probability is higher or lower, whereas IDI provides a measure of how far apart on average they are. NRI is based on a concept similar to IDI, but its focus is on the upward and downward movement of the predicted risks among those with and without events. NRI evaluates the “net” change in the proportion of subjects assigned a more appropriate risk or risk category (reclassification) using the new risk prediction model [20,21,22]. For study purposes, categorical NRI was computed according to clinically meaningful cut-points defining low, medium and high risk for MACE as follows: ≤0.47%, >0.47–0.70%, >0.7–0.93%, >0.93%. All statistical analyses were performed using the SAS software (version 9.4, Cary, NC, USA), with *p*-values < 0.05 considered as statistically significant.

## 3. Results

### 3.1. Clinical and Biological Phenotype

Overall, 82 ATTR-CA patients were enrolled: 13 (16%) with non-mutated TTR amyloidosis, 66 (80%) with variant TTR amyloidosis (47 with ATTR-V122I (p.Val142Ile), 9 with ATTR-I107V (p.Ile127Val), 2 with ATTR-V30M (p.Val50Met), 8 with other pathogenic transthyretin mutations), and 3 with undetermined genotypes (4%). The patients were predominantly male (89%), with a mean age of 70 ± 11 years at the time of spirometry/CPET testing (Table 1). A third of patients (33%) were in a non-sinusal rhythm, with respective frequencies of 23% and 11% for permanent atrial fibrillation and pacemaker implantations. All patients were under conventional treatment regimen for heart failure (angiotensin-converting enzyme (ACE) inhibitors, angiotensin receptor blockers (ARB), amiodarone, furosemide, anticoagulants). None were under beta-blockers, contra-indicated in cardiac amyloidosis patients.

Mean values for distinctive echocardiographic parameters were as follows: interventricular septum thickness 17 ± 4 mm, left ventricular ejection fraction 49 ± 15% and left atrial diameter 52 ± 11 mm. The ATTR biomarker staging system assessing disease severity classified more than half of the patients (57%) in Stages II and III (poorer prognosis). Median duration of patient follow-up was 9 months (interquartile range, IQR: 4–22), with 31 (38%) MACE observed among the study participants: 19 patients were hospitalized at least once due to heart failure and 12 died. MACE patients presented with higher disease severity (75% Stages II/III vs. 41% in “no MACE” patients; *p* = 0.01). Clinical, ECG, echocardiographic and biological parameters are further detailed in Table 1.

### 3.2. Cardiopulmonary Functional Phenotype

Lung volume restriction, evidenced by a restrictive spirometry pattern as per GLI/ERS predicted values, was observed in 49% of ATTR-CA patients (61% in “MACE” patients vs. 41% in “no MACE” patients; *p* = 0.08). Patients with a restrictive spirometry pattern presented with a characteristic severe rapid shallow breathing pattern illustrated by a higher respiratory frequency.

The analysis of the main functional features issuing from the pulmonary function and the cardiopulmonary exercise testing highlighted reduced resting lung volumes, as well as an impaired aerobic capacity in the MACE patients (Table 2). Mean peak aerobic capacity (pVO_2_) was 13.3 ± 3.3 mL.kg^−1^.min^−1^ (51% of predicted value) in the MACE patients compared with 16.1 ± 3.7 mL.kg^−1^.min^−1^ (67% of predicted value) in the patients with an absence of MACE. Forced vital capacity (FVC) was also decreased (71% vs. 82% predicted value) in the MACE patients, as was ventilatory reserve, with 35% of patients presenting with a ventilatory reserve under 25% (53% in “MACE” vs. 26% in “no MACE” patients; *p* = 0.06). Ventilatory inefficiency in the MACE patients was further ascertained by a high VEVCO_2_ slope of 42.6 ± 6.6. A value of VEVCO_2_ slope up to 35 was found in 95% of the MACE cases compared with 65% in the “no MACE” patients; *p* = 0.01.

### 3.3. Risk Profiles

Multivariate logistic regression indicated that an increased interventricular septum thickness, reduced FVC and impaired pVO_2_ maintained their significance as independent predictors of MACE (Table 3), with respective optimal Receiver Operating Characteristic (ROC) curve-derived cut-offs for FVC of 70% (Area Under the ROC Curve (95% Confidence Interval, AUC (95% CI): 0.67 (0.57–0.77); *p* < 0.01), and for pVO_2_ of 50% (AUC (95% CI): 0.72 (0.62–0.82); *p* < 0.01).

When MACE-free survival time was modelled over the follow-up period with Cox regression analysis (Table 4), only a reduced FVC < 70% predicted and a decreased pVO_2_ < 50% predicted were retained as independent factors, with respective Hazard Ratios (HR) of 7.01 (95% CI: 2.92–16.82; *p* < 0.01) and 2.48 (95% CI: 1.15–5.35; *p* = 0.02).

**Figure 1:**
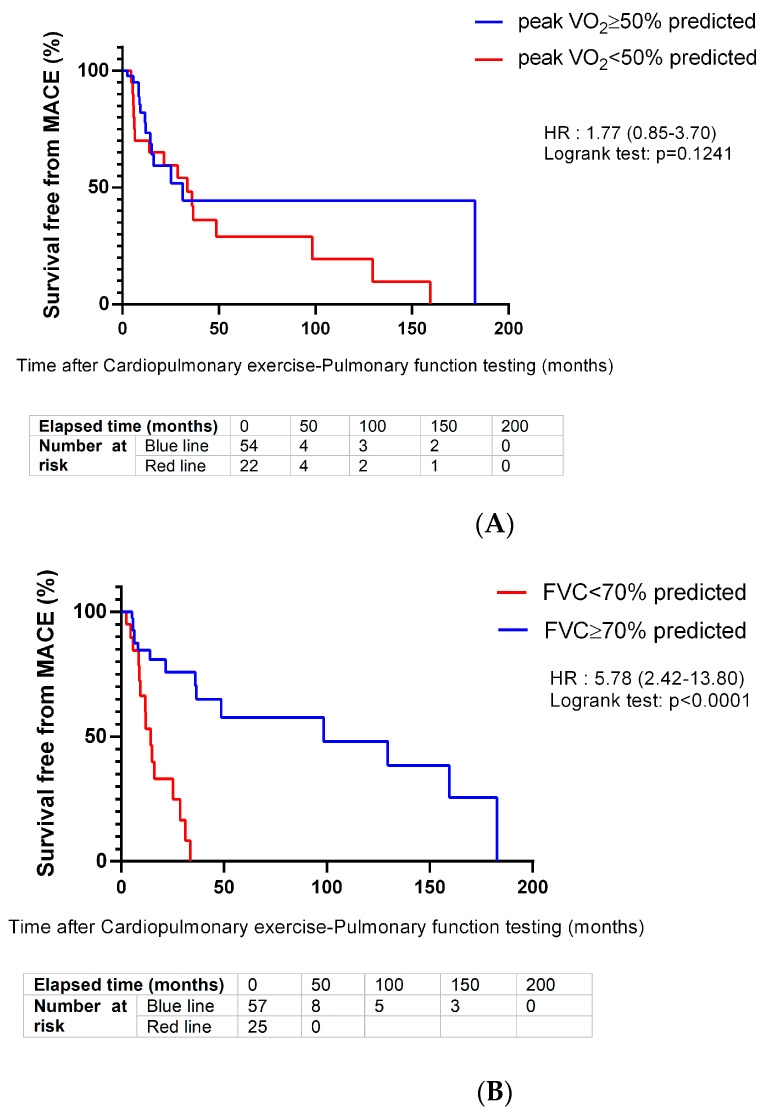

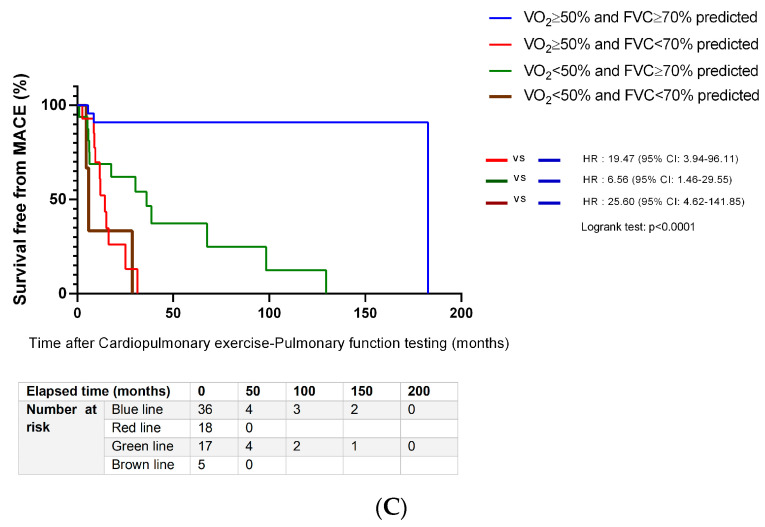
(**A**) Kaplan–Meier survival curves for MACE (composite of all-cause death or heart-failure-related hospitalization) with (A) peak VO_2_ (% predicted). Abbreviations: MACE: composite of heart-failure related hospitalization or all-cause death; peak VO_2_: peak oxygen uptake; FVC: forced vital capacity; HR: hazards ratio; *p*: *p*-value. Optimal cut-off points for peak VO_2_ and FVC determined by Receiver Operating Characteristic (ROC) curve analysis; statistical significance set at *p* < 5%. N.B: For each comparison group, the “Number at risk” table (below figure) presents, at distinct follow-up times (0, 50, 100, 150, 200 months), the number of patients at risk, i.e., the number of patients who have not yet experienced the event of interest (MACE) or censoring. (**B**) Kaplan–Meier survival curves for MACE (composite of all-cause death or heart-failure-related hospitalization) with FVC (% predicted). Abbreviations: MACE: composite of heart-failure-related hospitalization or all-cause death; peak VO_2_: peak oxygen uptake; FVC: forced vital capacity; HR: hazards ratio; *p*: *p*-value. Optimal cut-off points for peak VO_2_ and FVC determined by Receiver Operating Characteristic (ROC) curve analysis; statistical significance set at *p* < 5%. N.B: For each comparison group, the “Number at risk” table (below figure) presents, at distinct follow-up times (0, 50, 100, 150, 200 months), the number of patients at risk, i.e., the number of patients who have not yet experienced the event of interest (MACE) or censoring. (**C**) Kaplan–Meier survival curves for MACE (composite of all-cause death or heart-failure-related hospitalization) with the combination of peak VO_2_ and FVC. Abbreviations: MACE: composite of heart-failure-related hospitalization or all-cause death; peak VO_2_: peak oxygen uptake; FVC: forced vital capacity; HR: hazards ratio; *p*: *p*-value. Optimal cut-off points for peak VO_2_ and FVC determined by Receiver Operating Characteristic (ROC) curve analysis; statistical significance set at *p* < 5%. N.B: For each comparison group, the “Number at risk” table (below figure) presents, at distinct follow-up times (0, 50, 100, 150, 200 months), the number of patients at risk, i.e., the number of patients who have not yet experienced the event of interest (MACE) or censoring.

Figure 1A–C further illustrate the Kaplan–Meier curves of the primary endpoint MACE according to pVO_2_ (above and below 50% cut-off), FVC (above and below 70% cut-off) and the combination of pVO_2_ and FVC. Patients with reduced pVO_2_ (<50% predicted) presented with MACE more rapidly (mean survival free from MACE: 50 ± 13 months) compared with patients with pVO_2_ ≥ 50% (mean survival free from MACE: 90 ± 20 months) (log-rank *p*-value = 0.12) (Figure 1A).

The same tendency was observed for the patients with reduced FVC (<70% predicted) with the corresponding mean MACE-free survival of 16 ± 2 months compared with the patients with FVC ≥ 70% (mean survival free from MACE: 99 ± 16 months) (log-rank *p*-value < 0.01) (Figure 1B).

When pVO_2_ and FVC were considered simultaneously, three patient profiles were outlined, presenting with significantly differing risk profiles (*p* < 0.01) when compared to a referent patient group (pVO_2_ ≥ 50% and FVC ≥ 70%) with a mean survival of 167 ± 13 months (Figure 1C).

The highest risk group consisted of patients presenting with both impaired pVO_2_ (<50%) and FVC (<70%), with an HR for MACE of 25.60 (95% CI: 4.62–141.85, *p* < 0.01). Patients with the highest risk presented with a mean survival of 15 ± 6 months, and were thus subject to MACE onset, on average, 9–13 years earlier compared to patients with the lowest risk (referent group).

### 3.4. Prognostic Value of Impaired pVO_2_ and FVC

**Figure 2:**
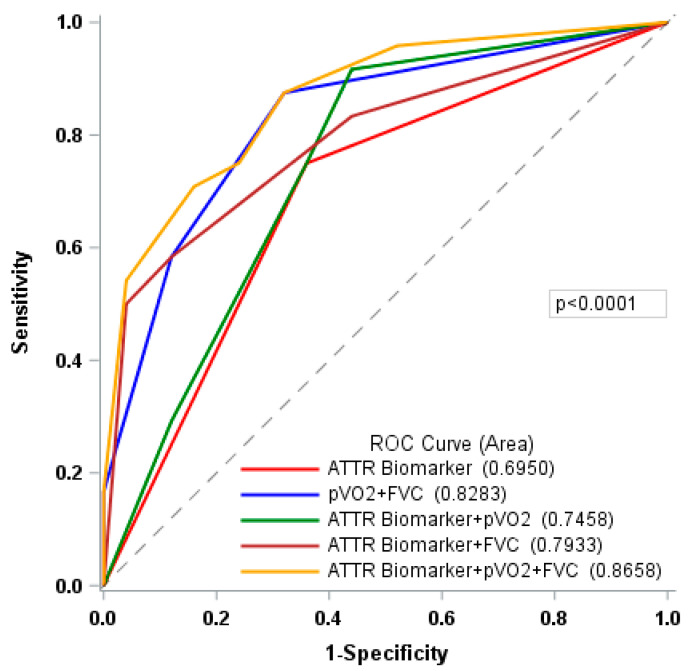
Receiver Operating Characteristic (ROC) contrast for ATTR biomarker staging, peak VO_2_ (% predicted) and FVC (% predicted). Reference curve: ATTR biomarker. Abbreviations: ATTR: transthyretin amyloidosis; pVO_2_: peak oxygen uptake (cut-off: 50%); FVC: forced vital capacity (cut-off:70%); AUC: area under the ROC curve; Interpretation: 0.7 ≤ AUC < 0.8: acceptable discrimination; 0.8 ≤ AUC < 0.9: excellent discrimination; 0.9 ≤ AUC ≤ 1: perfect discrimination.

ROC analysis (Figure 2) further sustained a good performance of pVO_2_ (cut-off: 50%) and FVC (cut-off: 70%) in discriminating MACE risk (AUC: 0.83 (95% CI: 0.72–0.94)) compared with Gillmore’s validated biomarker staging score for ATTR (AUC: 0.70 (95% CI: 0.56–0.83)). It is to be further noted that combined with the ATTR biomarker staging score, FVC’s discriminating capacity (AUC: 0.79 (95% CI: 0.67–0.92)) seems to exceed that of PVO_2_ (AUC: 0.75 (95% CI: 0.61–0.88)). The best performance was achieved by the combination of all three parameters (ATTR biomarker staging, pVO_2_ (cut-off: 50%, FVC (cut-off:70%)), with a significantly improved prediction of MACE (AUC: 0.87 (95% CI: 0.77–0.96); *p* < 0.01).

Time-dependent ROC analysis yielded the same tendencies, with suitable goodness-of-fit for models integrating both FVC and pVO_2_: Harrell’s C-index > 0.7 (Table 5).

**Figure 3:**
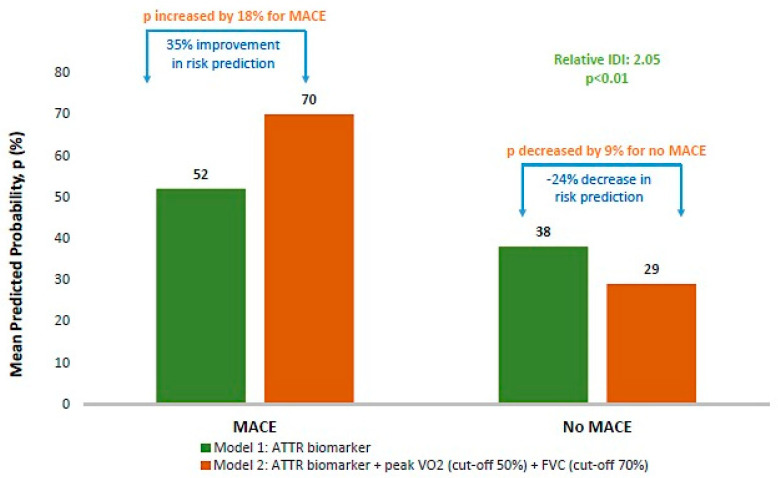
Integrated Discrimination Improvement (IDI) for ATTR biomarker staging, peak VO_2_ (% predicted) and FVC (% predicted). Abbreviations: MACE: composite of heart-failure-related hospitalization or all-cause death; IDI: integrated discrimination improvement; *p*: mean predicted probability; ATTR: transthyretin amyloidosis; peak VO_2_: peak oxygen uptake; FVC: forced vital capacity. Optimal cut-off points for peak VO_2_ and FVC determined by Receiver Operating Characteristic (ROC) curve analysis; statistical significance set at *p* < 5%. Calculation details: (1) variation of mean predicted probability, *p*: (p_Model 2_ − p_Model 1_); (2) variation in risk prediction: [(p_Model 2_ − p_Model 1_)/p_Model 1_].

The quantification of the added predictive value of the functional parameters FVC (cut-off: 70%) and pVO_2_ (cut-off: 50%) was in line with the above results. Indeed, the simultaneous consideration of FVC, pVO_2_ and ATTR biomarker staging led to a significant average improvement of 35% in MACE risk prediction (Figure 3) compared to ATTR biomarker staging alone.

**Figure 4:**
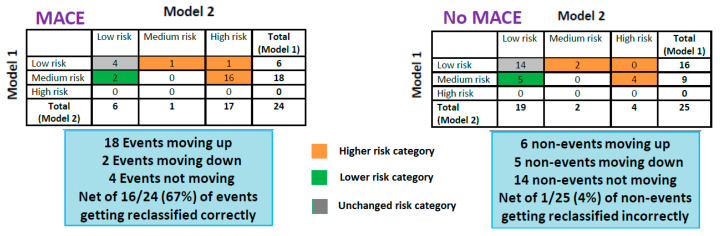
Net Reclassification Improvement (NRI) for ATTR biomarker staging, peak VO_2_ (% predicted) and FVC (% predicted). Model 1: ATTR biomarker; Model 2: ATTR biomarker + peak VO_2_ (cut-off 50%) + FVC (cut-off 70%); Event defined as MACE; non-event defined as “no MACE”. Abbreviations: MACE: composite of heart-failure-related hospitalization or all-cause death; ATTR: transthyretin amyloidosis; peak VO_2_: peak oxygen uptake; FVC: forced vital capacity; NRI: Net Reclassification Improvement. Optimal cut-off points for peak VO_2_ and FVC determined by Receiver Operating Characteristic (ROC) curve analysis. Table Interpretation (e.g., MACE table): (1) first row: 6 “low-risk” patients (Model 1) are reclassified by Model 2 as follows: 4 low-risk, 1 medium-risk and 1 high-risk. As such, 4 patients with MACE (events) do not move, and 2 events move up to higher risk categories; (2) second row: 18 “medium-risk” patients (Model 1) are reclassified by Model 2 as follows: 2 events move down to the low risk category, 16 patients move up to the high risk category, 0 patients remain in the medium risk category; (3) overall, Model 2 reclassifies 18 patients to higher risk categories and 2 patients to lower risk categories (4 patients with unchanged risk categories); (4) Event NRI = ((18 − 2)/24) ∗ 100 = 67%; (5) Non-Event (Non-MACE) NRI = ((6 − 5)/25) ∗ 100 = 4%; (6) Overall NRI = (67% − 4%) = 63%.

The resultant was the correct reclassification of 63% of the overall study population into more appropriate risk groups (*p* < 0.01), with 67% of the MACE patients reassigned a higher risk category (*p* < 0.01; Hosmer–Lemeshow goodness-of-fit test: *p* = 0.95) (Figure 4).

## 4. Discussion

With the advent of novel therapies for transthyretin cardiac amyloidosis, the identification of pertinent prognostic strategies with the most accurate ability to detect increased adverse event risk in patients is an important research pursuit. While clinical, echocardiographic and biological parameters are traditional severity markers in this chronic disease population, it has also been highlighted that impaired peak VO_2_ (pVO_2_) detains prognostic value, which might be optimized when considered with another functional parameter, lung volume restriction. We thus deemed that determining the potential ability of the combination of impaired pVO_2_ and lung volume restriction to provide superior prognostic resolution would be a viable research endeavor, particularly compared with other established prognostic markers, such as ATTR biomarker staging.

Novel findings of our study are the following. Firstly, our study suggests that reduction in lung volume is a common feature in ATTR-CA patients (49%). Secondly, a restrictive spirometry pattern (FVC < 70% predicted) and reduced pVO_2_ (<50% predicted) are identified as independent predictors of poor outcome (MACE), defined as either all-cause death or heart-failure-related hospitalization in ATTR-CA patients. MACE-free survival time significantly declines in patients with reduced FVC and impaired pVO_2_, with MACE onset occurring, on average, 9 to 13 years earlier compared to patients with a normal spirometry pattern and normal aerobic capacity. Thirdly, the combined use of spirometry (FVC) and pVO_2_ improves risk stratification based on biomarker staging (NT-proBNP levels and estimated glomerular filtration rate) of ATTR-CA patients, with a 35% improvement in patient risk discrimination and 67% of the MACE patients reassigned into a higher risk category. IDI and NRI analyses further suggest that the greatest benefit in combining these three severity markers is moving patients with MACE into a higher risk group.

In accordance with earlier studies, we thus confirmed that reduced pVO_2_ and increased VEVCO_2_ slope seem to be strongly and independently predictive of MACE in ATTR-CA patients [5,6,7,9,10]. In addition, we also found that the presence of a restrictive spirometry pattern was associated with increased MACE risk in ATTR-CA, consistent with other authors reporting that the presence of a restrictive ventilatory pattern is predictive of all-cause and cardiovascular mortality [11,12,13]. Indeed, spirometry parameters can predict outcomes and improve risk stratification based on pVO_2_ in chronic heart failure patients with reduced ejection fraction [13].

Pulmonary manifestations rarely dominate the clinical picture of ATTR cardiac amyloidosis [23,24]. In this amyloidosis type, lung involvement is most often a post mortem finding, as evidenced by diffuse amyloid deposit in alveolar septal and vessel walls [24,25]. However, some lines of evidence support the assertion that pulmonary involvement may be diagnosed ante mortem [23,26,27], with bone tracer scintigraphy and chest computed tomography commonly displaying abnormal pulmonary imaging [28,29,30]. In spite of the absence of clinically overt pulmonary involvement, a reduction in lung volume has been consistently reported using pulmonary functional parameters in ATTR-CA patients. In the present study, restrictive spirometry pattern was observed along with rapid and shallow breathing and ventilatory inefficiency during physical exertion. Moreover, it is important to note that the mechanisms of lung restriction often described in patients with heart failure [28,29] are not readily evident in ATTR-CA patients. No signs of lung edema, pleural effusion or increased cardiac size were observed in our patients. Nonetheless, our findings unequivocally suggest that behind the purported relative clinical silence, a restrictive spirometry pattern might be of certain clinical importance in ATTR-CA as corroborated by the observed significant association between MACE survival and lung volume restriction in our study population, as well as the high discriminating capacity of reduced forced vital capacity. Indeed, an optimal cutoff value for FVC of less than 70% of its predicted value defined a high to very high risk of MACE onset in our patients.

### Study Limitations

An obvious limitation of the present investigation is its retrospective design, as well as the relatively small number of MACE events. Only 82 patients (31 events) were included, thus possibly resulting in a potential lack of statistical power to detect other potential factors with moderate effects on outcome. This small sample size is mainly explained by the fact that cardiac amyloidosis is a rare disease even in expert centers evaluating cardiopulmonary function in CA patients. Plethysmography was not available in all ATTR-CA patients. Plethysmography requires special features that are not available on a metabolic cart with VO_2_ testing equipment. Hence, due to the lack of routine availability of plethysmography, advanced pulmonary function testing was not used to confirm our interpretations of airflow and ventilatory patterns resulting from basic spirometry testing. In contrast, spirometry remains one of the simplest and most widely available methods to assess pulmonary function.

## 5. Conclusions

To the best of our knowledge, this is the first exploratory study of the kind, assessing the combined prognostic value of pVO_2_, FVC and ATTR biomarker staging. The unequivocal changes in the three metric measures generally used to assess the predictive ability of a marker (AUC, IDI, NRI) all underline that combining biological (ATTR biomarker staging) and functional markers (pVO_2_ and FVC) synergistically improved prognostic resolution in our cohort of patients with transthyretin cardiac amyloidosis. Our results suggest that the greatest benefit in combining these three severity markers is moving patients with adverse outcome (MACE) into a higher risk group, with the identification of subjects at greatest MACE risk with significantly greater accuracy compared with either marker alone. This additive predictive capacity of the three combined markers was consistent across our rather heterogeneous study population in terms of genetic variability (wild-type ATTR, different pathogenic ATTR variants) and disease severity (mild to severe disease states ascertained by the NYHA classification and ATTR biomarker staging). Our results need to be confirmed by future prospective investigations with a larger number of events, allowing for a more powerful multivariable analysis taking into account negative multisystem impact (i.e., cardiovascular, autonomic, pulmonary, renal, and skeletal muscle) of ATTR-CA. Such a multivariable scoring system would dramatically improve the ability to portend adverse event risk and allow more comprehensive risk prediction in these patients.

In light of the potential highly informative capacity of CPET and spirometry in predicting adverse outcome in ATTR-CA patients, these tests might prove to be highly useful tools, significantly contributing towards improved risk prediction, optimized patient monitoring and clinical decision making, as well as the timely introduction of newer-generation anti-amyloid therapies.

## Figures and Tables

**Table 1:** Baseline characteristics of ATTR cardiac amyloidosis patients according to outcome (heart-failure-related hospitalization or all-cause death).

	All Patients (n = 82)	No MACE (n = 51)	MACE (n = 31)	*p*-Value
Age, years	70 ± 11	71 ± 11	68 ± 10	0.258
Sex (Male gender)	73 (89)	45 (88)	28 (90)	1.000
BMI, kg/m^2^	26 ± 4	26 ± 5	25 ± 4	0.497
Type 2 Diabetes	15 (18)	10 (20)	5 (16)	0.693
Hypertension	37 (45)	19 (37)	18 (58)	0.066
Carpal tunnel	46 (56)	30 (59)	16 (52)	0.524
NYHA III/IV n = 77	52 (68)	29 (62)	23 (77)	0.171
Non-sinusal rhythm	27 (33)	13 (25)	14 (45)	0.066
ECG/Echocardiography				
QS wave n = 72	19 (26)	10 (23)	9 (31)	0.463
Low QRS voltage n = 78	11 (14)	6 (12)	5 (17)	0.738
IVS thickness, mm n = 79	17 ± 4	16 ± 3	19 ± 3	<0.001
LVEDV, mL n = 61	101 ± 40	101 ± 43	100 ± 36	0.478
LVMI, g/m^2^ n = 74	170 ± 52	154 ±51	194 ± 45	0.002
LVEF, % n = 80	49 ± 15	51 ± 15	46 ± 15	0.106
Cardiac index, L/min/m^2^ n = 71	2.0 ± 0.5	2.1 ± 0.5	1.9 ± 0.4	0.245
LA diameter, mm n = 41	52 ± 11	52 ± 11	53 ± 10	0.448
E/Ea n = 47	2 ± 2	2 ± 2	2 ± 1	0.479
E/e’ ratio n = 72	16 ± 7	14 ± 6	18 ± 8	0.095
TDE, msec n = 56	156 ± 41	154 ± 43	157 ± 38	0.419
Systolic PAP, mmHg n = 30	38 ± 12	37 ± 12	40 ± 11	0.595
TAPSE, mm n = 28	18 ± 4	19 ± 4	18 ± 6	1.000
Biological parameters				
eGFR, mL/min/1.72 m^2^ n = 78	69 [9–169]	73 [11–169]	55 [9–162]	0.103
Cardiactroponin T, ng/L n = 60	73 [7–566]	58 [7–376]	109 [22–566]	0.030
NT-proBNP, ng/L n = 53	2684 [50–15,770]	1736 [50–5766]	3442 [1154–15,770]	0.004
ATTR Biomarker staging II + III vs. I n = 53 *	30 (57) vs. 23 (43)	12 (41) vs. 17 (59)	18 (75) vs. 6 (25)	0.014

Abbreviations: Age: age at spirometry/CPET; MACE: composite of all-cause death or heart failure-related hospitalization; CPET: Cardio Pulmonary Exercise testing; ATTR: transthyretin amyloidosis; BMI: body mass index; NYHA: New York Heart Association (NYHA) classification; ECG: electrocardiogram; IVS: interventricular septum thickness; LVEDV: left ventricle end diastolic volume; LVMI: Left Ventricle Mass Index; LVEF: left ventricular ejection fraction; LA: left atrium; E/Ea; peak of pulsed Doppler E wave/average peak of annulus tissue Doppler imaging E’ waves; TDE: E deceleration time; PAP: pulmonary arterial pressure; TAPSE: tricuspid annular systolic excursion; eGFR: estimated glomerular filtration rate; NT-proBNP: N-terminal pro B-type Natriuretic Peptide. * ATTR Biomarker staging: Stage I: NT-proBNP ≤ 3000 ng/L and eGFR ≥ 45 mL/min, Stage III: NT-proBNP > 3000 ng/L and eGFR < 45 mL/min, Stage II: the remainder [15]. Results are presented as mean ± standard deviation or median and interquartile ranges (IQR 25–75%) for quantitative variables, and as absolute value or absolute value (percentage) for categorical variables; statistical significance set at *p* < 0.05.

**Table 2:** Spirometry and cardiopulmonary exercise variables in ATTR cardiac amyloidosis patients according to outcome (heart-failure-related hospitalization or all-cause death).

	All Patients (n = 82)	No MACE (n = 51)	MACE (n = 31)	*p*-Value
FEV1, % predicted *	76.6 ± 17.0	80.8 ± 15.2	69.8 ± 17.8	0.038
FVC, % predicted *	77.8 ± 17.4	82.0 ± 15.5	70.8 ± 18.4	0.113
FVC < 70 % predicted ^†^	25 (30.5)	9 (17.7)	16 (51.6)	0.001
FEV1/FVC, % n = 80	77.7 ± 9.4	77.4 ± 10.2	78.2 ± 8.0	1.000
TLC, % predicted * n = 41	75.8 ± 16.3	75.1 ± 17.4	78.8 ± 10.8	0.939
DLCO, % n = 40	78.0 ± 17.6	76.3 ± 19.0	81.5 ± 14.6	0.732
Peak workload, watts n = 53	75.8 ± 30.3	80.1 ± 32.1	66.8 ± 24.2	0.164
Peak workload, % n = 49	56.4 ± 19.2	59.4 ± 18.7	50.3 ± 19.3	0.087
Peak VO_2_, mL.kg^−1^.min^−1^ n = 74	15.0 ± 3.8	16.1 ± 3.7	13.3 ± 3.3	0.001
Predicted peak VO_2_, % n = 76	60.5 ± 18.9	67.0 ± 17.6	51.0 ± 16.6	<0.001
Peak VO_2_ < 50% predicted ^†^ n = 76	22 (29.0)	5 (11.1)	17 (54.8)	<0.001
ATVO_2_, mL.kg^−1^.min^−1^ n = 48	11.1 ± 3.2	11.6 ± 3.5	10.1 ± 2.4	0.069
Peak VO_2_/watt slope, mL.watt^−1^ n = 53	10.2 ± 2.4	9.6 ± 1.9	11.5 ± 3.0	0.333
Peak RER n = 75	1.2 ± 0.1	1.2 ± 0.1	1.2 ± 0.2	0.561
Peak VE/VO_2_ n = 67	45.1 ± 10.1	43.1 ± 9.7	47.7 ± 10.2	0.019
Peak VE/VCO_2_ n = 67	38.5 ± 6.6	37.4 ± 6.2	40.1 ± 7.0	0.277
Peak BF, min^−1^ n = 67	37.6 ± 16.8	36.2 ± 9.9	39.6 ± 23.4	0.552
Peak Vt/FVC, % n = 65	51.6 ± 14.3	51.7 ± 13.4	51.4 ± 15.8	0.394
Ventilatory reserve, % n = 51	31.5 ± 18.4	34.3 ± 17.6	25.8 ± 19.1	0.170
VE VCO_2_ slope n = 62	39.2 ± 7.8	37.7 ± 7.9	42.6 ± 6.6	0.003
Peak O_2_ pulse, % n = 51	72.9 ± 19.1	74.1 ± 20.3	70.5 ± 16.8	0.170
Systolic pressure at rest, mmHg n = 68	128.4 ± 23.6	129.7 ± 18.4	126.7 ± 29.4	0.465
Peak systolic pressure, mmHg n = 73	156.2 ± 38.5	162.4 ± 34.8	147.4 ± 42.2	0.187
Diastolic pressure at rest, mmHg n = 68	80.3 ± 14.8	80.6 ± 12.7	79.8 ± 17.5	0.687
Peak diastolic pressure, mmHg n = 73	86.1 ± 22.4	87.2 ± 21.6	84.5 ± 23.9	0.187
Heart rate at rest, bpmn = 75	79.9 ± 13.4	80.2 ± 14.2	79.6 ± 12.3	0.793
Peak heart rate, bpmn = 75	122.2 ± 21.3	123.5 ± 22.6	120.4 ± 19.4	0.703
Peak heart rate, % maximal pred. n = 75	81.1 ± 13.4	82.2 ± 13.2	79.4 ± 13.8	0.925
Heart rate reserve used, % n = 75	61.5 ± 26.1	64.2 ± 25.8	57.6 ± 26.4	0.708

Abbreviations: MACE: composite of all-cause death or heart-failure-related hospitalization; FEV1: forced expiratory volume in 1 s; FVC: forced vital capacity; TLC: Total Lung Capacity; DL_CO_: diffusing capacity for carbon monoxide; VO_2_: oxygen uptake; AT: anaerobic threshold; RER: respiratory exchange ratio; VE: minute ventilation; VCO_2_: pulmonary carbon dioxide output; BF: breathing frequency; Vt: tidal volume; O_2_: oxygen; bpm: beat per minute; GLI: Global Lung Function Initiative. * presented as percent-of-predicted normal FEV1 and FVC values. Predicted normal values are average GLI predicted values for FEV1 and FVC in the population for any person of similar age, sex, body composition and race [17,18]. ^†^ optimal cut-off points determined by Receiver Operating Characteristic (ROC) Curve analysis. Results are presented as mean ± standard deviation. Statistical significance was set at *p* < 0.05.

**Table 3:** Predictors of heart-failure-related hospitalization or all-cause death (MACE) in ATTR cardiac amyloidosis patients: univariate and multivariate logistic regression analysis (n = 82).

	Univariate Analysis		Multivariate Analysis	
	OR (95% CI)	*p*-Value	OR (95% CI)	*p*-Value
Age at CPET-PFT, years	0.98 (0.94–1.02)	0.32		
Male gender	1.24 (0.29–5.38)	0.77		
BMI kg/m^2^	0.97 (0.87–1.07)	0.50		
NYHA III/IV	2.04 (0.73–5.71)	0.18		
Non-sinusal rhythm, %	2.41 (0.93–6.21)	0.07		
Carpal tunnel, %	0.75 (0.30–1.83)	0.52		
Hypertension, %	2.33 (0.94–5.80)	0.07		
Diabetes, %	0.79 (0.24–2.57)	0.69		
eGFR, mL/min/1.72 m^2^	0.98 (0.97–1.00)	0.02 ^‡^		
Cardiac troponin T, ng/L	1.006 (1.002–1.011)	0.01 ^‡^		
NT-proBNP, ng/L	1.001 (1.000–1.001)	<0.01 ^‡^		
ATTR Biomarker staging: Stage II + III vs. Stage I *	4.25 (1.30–13.87)	0.02 ^‡^		
IVS thickness, mm	1.26 (1.09–1.46)	<0.01 ^‡^	1.23 (1.00–1.50)	0.05
LVEF, %	0.98 (0.95–1.01)	0.15		
Systolic PAP, mmHg	1.02 (0.95–1.09)	0.58		
FEV1, % predicted ^†^		<0.01 ^‡^		
<70	4.38 (1.60–11.98)			
≥70	reference			
FVC, % predicted ^†^		<0.01 ^‡^	16.17 (3.47–75.48)	<0.01
<70	4.98 (1.82–13.63)			
≥70	reference			
Peak VO_2_, % predicted ^†^		<0.01 ^‡^	18.27 (3.73–89.48)	<0.01
<50	9.71 (3.02–31.24)			
≥50	reference			
Ventilatory reserve, %	0.97 (0.94–1.01)	0.12		
VE VCO_2_ slope	1.09 (1.01–1.18)	0.03 ^‡^		
Peak O_2_ pulse, %	0.99 (0.96–1.02)	0.53		
Peak heart rate, % maximal predicted	0.99 (0.95–1.02)	0.39		
Heart rate reserve used, %	0.99 (0.97–1.01)	0.28		

Abbreviations: MACE: composite of all-cause death or heart-failure-related hospitalization; ATTR: transthyretin amyloidosis; OR: odds ratio; CI: Confidence Interval; CPET: Cardiopulmonary Exercise Testing; PFT: Pulmonary Function Testing; BMI: body mass index; NYHA: New York Heart Association (NYHA) classification; eGFR: estimated glomerular filtration rate; NT-proBNP: N-terminal pro B-type Natriuretic Peptide; IVS: interventricular septum thickness; LVEF: Left Ventricular Ejection Fraction; PAP: pulmonary arterial pressure; FEV_1_: forced expiratory volume in 1 s; FVC: forced vital capacity; VO_2_: oxygen uptake; VE: minute ventilation; VCO_2_, pulmonary carbon dioxide output: O_2_: Oxygen. * ATTR Biomarker staging: Stage I: NT-proBNP ≤ 3000 ng/L and eGFR ≥ 45 mL/min, Stage III: NT-proBNP > 3000 ng/L and eGFR <45 mL/min, Stage II: the remainder [15]. ^†^ optimal cut-off points determined by Receiver Operating Characteristic (ROC) Curve analysis. ^‡^ Variables with significant association in univariate logistic regression analysis (* *p* < 0.05) were considered for multivariate analysis. Variables entered into the initial multivariate model for MACE: cardiac troponin T, ATTR Biomarker staging, interventricular septum thickness, FVC and peak VO_2_. Statistical significance level for multivariate analysis set at *p* < 0.05. Goodness-of-fit of final multivariate model: *p* = 0.7019 (Hosmer–Lemeshow test); AUC-ROC curve = 0.8886 (95% CI: 0.817–0.960), *p* < 0.0001).

**Table 4:** Cox regression analysis of survival free from heart-failure-related hospitalization or all-cause death (MACE) in ATTR cardiac amyloidosis patients (n = 82).

	Univariate Analysis		Multivariate Analysis	
	HR (95% CI)	*p*-Value	HR (95% CI)	*p*-Value
Age, years	1.04 (1.00–1.08)	0.07 ^‡^		
Male gender	1.37 (0.32–5.89)	0.67		
BMI kg/m^2^	0.92 (0.83–1.02)	0.09 ^‡^		
NYHA III/IV	0.74 (0.31–1.76)	0.49		
Non-sinusal rhythm,%	1.52 (0.74–3.13)	0.25		
Hypertension, %	0.67 (0.31–1.44)	0.31		
Type 2 diabetes, %	2.34 (0.87–6.35)	0.09 ^‡^		
eGFR, mL/min/1.72 m^2^	1.00 (0.99–1.01)	0.78		
Cardiactroponin T, ng/L	1.00 (1.00–1.01)	0.06 ^‡^		
NT-proBNP, ng/L	1.00 (1.00–1.00)	0.02 ^‡^		
ATTR Biomarker staging: Stage II + III vs. Stage I *	1.66 (0.65–4.24)	0.29		
IVS thickness, mm	1.04 (0.94–1.16)	0.42		
LVEF, %	0.99 (0.97–1.02)	0.55		
Systolic PAP, mmHg	0.99 (0.94–1.05)	0.79		
FEV_1_, % predicted ^†^		0.01 ^‡^		
<70	2.58 (1.20–5.54)			
≥70	reference			
FVC, % predicted ^†^		<0.01 ^‡^	7.01 (2.92–16.82)	<0.01
<70	5.78 (2.42–13.80)			
≥70	reference			
PeakVO_2_, % predicted ^†^		0.13 ^‡^	2.48 (1.15–5.35)	0.02
<50	1.77 (0.85–3.70)			
≥50	reference			
Ventilatory reserve, %	0.99 (0.95–1.02)	0.37		
VE VCO_2_ slope	1.05 (1.00–1.11)	0.05 ^‡^		
Peak O_2_ pulse, %	0.99 (0.96–1.01)	0.32		
Peak heart rate, % maximal predicted	1.00 (0.97–1.03)	0.82		
Heart rate reserve used, %	1.00 (0.99–1.01)	0.95		

Abbreviations: Age: age at spirometry/CPET; MACE: composite of all-cause death or heart-failure-related hospitalization; ATTR: transthyretin amyloidosis; OR: odds ratio; CI: Confidence Interval; CPET: Cardiopulmonary Exercise Testing; BMI: body mass index; NYHA: New York Heart Association (NYHA) classification; eGFR: estimated glomerular filtration rate; NT-proBNP: N-terminal pro B-type Natriuretic Peptide; IVS: interventricular septum thickness; LVEF: Left Ventricular Ejection Fraction; PAP: pulmonary arterial pressure; FEV_1_: forced expiratory volume in 1 s; FVC: forced vital capacity; VO_2_: oxygen uptake; VE: minute ventilation; VCO_2_, pulmonary carbon dioxide output: O_2_: Oxygen. * ATTR Biomarker staging: Stage I: NT-proBNP ≤ 3000 ng/L and eGFR ≥ 45 mL/min, Stage III: NT-proBNP > 3000 ng/L and eGFR < 45 mL/min, Stage II: the remainder [15]. _†_ optimal cut-off points determined by Receiver Operating Characteristic (ROC) Curve analysis. ^‡^ Variables with significant association in univariate analysis (* *p* < 0.15) were considered for multivariate analysis. Variables entered into the initial multivariate Cox model for MACE: age at spirometry/CPET, BMI, type 2 diabetes, cardiac troponin T, NT-proBNP, FVC and peak VO_2_. Statistical significance level set at *p* < 0.05.

**Table 5:** Time-dependent ROC analysis of predictors of heart-failure-related hospitalization or all-cause death (MACE) in ATTR cardiac amyloidosis patients (n = 82).

	Harrell’s C-Index	Integrated AUC
ATTR Biomarker	0.5675	0.4341
pVO_2_ + FVC	0.7228	0.6769
ATTR Biomarker + pVO_2_	0.6127	0.4775
ATTR Biomarker + FVC	0.6393	0.5265
ATTR Biomarker + pVO_2_ + FVC	0.7039	0.5770

Abbreviations: ROC: receiver operating characteristic; ATTR: transthyretin amyloidosis; C-index: concordance index; AUC: area under the ROC curve; pVO_2_: peak oxygen uptake; FVC: forced vital capacity. ATTR Biomarker staging: Stage I: NT-proBNP ≤ 3000 ng/L and eGFR ≥ 45 mL/min, Stage III: NT-proBNP > 3000 ng/L and eGFR < 45 mL/min, Stage II: the remainder. Optimal cut-off points for pVO_2_ and FVC determined by ROC analysis: pVO_2_ (cut-off 50%); FVC (cut-off 70%). Interpretation of AUC (discrimination) and Harrell’s C-index (calibration): 0.7 ≤ AUC < 0.8: good discrimination; 0.8 ≤ AUC < 0.9: excellent discrimination; 0.9 ≤ AUC ≤ 1: perfect discrimination; 0.7 ≤ Harrell’s C-index < 0.8: good calibration; 0.8 ≤ Harrell’s C-index < 0.9: excellent calibration; 0.9 ≤ Harrell’s C-index ≤ 1: perfect calibration.

## Data Availability

Data are available from Pr NEVIERE Remi on reasonable request.

## Abbreviations

AL	light-chain amyloidosis
AT	anaerobic threshold
ATTR-CA	transthyretin cardiac amyloidosis
ATTRwt	non-mutated transthyretin
ATTRv	variant transthyretin
AUC	area under the ROC curve
BF	breathing frequency
BMI	body mass index
bpm	beat per minute
BF	breathing frequency
CI	confidence interval
CPET	Cardio Pulmonary Exercise testing
DLCO	diffusing capacity for carbon monoxide
ECG	Electrocardiogram
E/Ea	peak of pulsed Doppler E wave/average peak of annulus tissue Doppler imaging E’ waves
eGFR	estimated glomerular filtration rate
ECG	electrocardiogram
ERS	European Respiratory Society
FEV1	forced expiratory volume in the first second
FVC	forced vital capacity
GLI	global lung function initiative
HR	heart rate OR hazards ratio (resulting from Cox regression analysis)
hs	high sensitivity
IQR	interquartile range
IDI	integrated discrimination improvement
IRB	institutional review board
IVS	interventricular septum thickness
LA	left atrium
LVEF	left ventricular ejection fraction
LVEDV	left ventricle end diastolic volume
LVMI	Left Ventricle Mass Index
MACE	composite of all-cause death or heart-failure-related hospitalization
MVV	maximal voluntary ventilation
NRI	net reclassification improvement
NT-proBNP	N-terminal pro-B-type Natriuretic Peptide
NYHA	New York Heart Association (NYHA) classification
O2 pulse	peak oxygen pulse
PAP	pulmonary arterial pressure
PFT	Pulmonary Function Testing
pVO2	peak aerobic capacity
RER	respiratory exchange ratio
ROC	receiver operating characteristic
TAPSE	tricuspid annular systolic excursion
TDE	E deceleration time
TLC	Total Lung Capacity
VE	minute ventilation
VCO2	pulmonary carbon dioxide output
VO2	oxygen uptake
Vt	tidal volume
